# Systemic Mastocytosis: Following the Tyrosine Kinase Inhibition Roadmap

**DOI:** 10.3389/fphar.2020.00443

**Published:** 2020-04-14

**Authors:** Miguel Piris-Villaespesa, Ivan Alvarez-Twose

**Affiliations:** ^1^Servicio de Hematología y Hemoterapia and IRYCIS, Hospital Universitario Ramón y Cajal, Madrid, Spain; ^2^Instituto de Estudios de Mastocitosis de Castilla La Mancha (CLMast) and CIBERONC, Hospital Virgen del Valle, Toledo, Spain

**Keywords:** mast cell, systemic mastocytosis, tyrosine kinase inhibitor, KIT, imatinib, midostaurin, avapritinib, cytoreductive therapy

## Abstract

Systemic mastocytosis is a rare and heterogeneous disease characterized by mast cell proliferation and activation. KIT is a transmembrane tyrosine kinase which plays a key role in mast cell growth, differentiation and survival. After interaction with its ligand, the stem cell factor, KIT dimerizes activating downstream pathways involving multiple tyrosine kinases (PI3K, JAK/STAT, RAS/ERK). Activating mutations in KIT are detected in most cases of systemic mastocytosis, being the most common *KIT* D816V. Therefore, since the emergence of tyrosine kinase inhibitors, KIT inhibition has been an attractive approach when facing mastocytosis treatment. Initial reports showed that only the rare *KIT* D816V negative cases were responsive to tyrosine kinase inhibitors. However, the development of new tyrosine kinase inhibitors such as midostaurin or avapritinib with activity against mast cells carrying the D816V KIT mutation, has changed the landscape of this disease.

## Introduction

Systemic mastocytosis (SM) is a heterogeneous disease characterized by a clonal expansion and accumulation of neoplastic mast cells (MCs) in cutaneous and/or extracutaneous organs (Arber et al., 2016). The stem cell factor (SCF) receptor KIT is a transmembrane tyrosine kinase (TK) protein codified by the *KIT* oncogene that plays a key role in the function of MCs, *via* regulation of their differentiation, maturation, migration, survival, and cytokine production (Cruse et al., 2014). Mutations involving the activating domain of *KIT*, mostly the D816V *KIT* mutation, are found in >90% of patients with SM when highly-sensitive diagnostic techniques are used (Garcia-Montero et al., 2006; Kristensen et al., 2014; Jara-Acevedo et al., 2015).This mutation leads to a constitutive SCF-independent activation of the receptor (Orfao et al., 2007; Arock et al., 2015) favoring downstream signaling intracellular pathways that promote MC proliferation, growth, survival and activation (Cruse et al., 2014). Since the discovery of the pathogenic role of *KIT* in SM, many investigations have been focused on the treatment of SM patients with TK inhibitors (TKIs). Although early studies showed that only the rare D816V-negative cases were sensitive to imatinib, new TKIs have shown inhibitory activity also against MCs carrying the D816V *KIT* mutation, which has expanded the current therapeutic landscape in SM. This review is focused on the role of TKI drugs in the management of SM.

### KIT in Normal Mast Cells and in Mastocytosis

**Figure 1:**
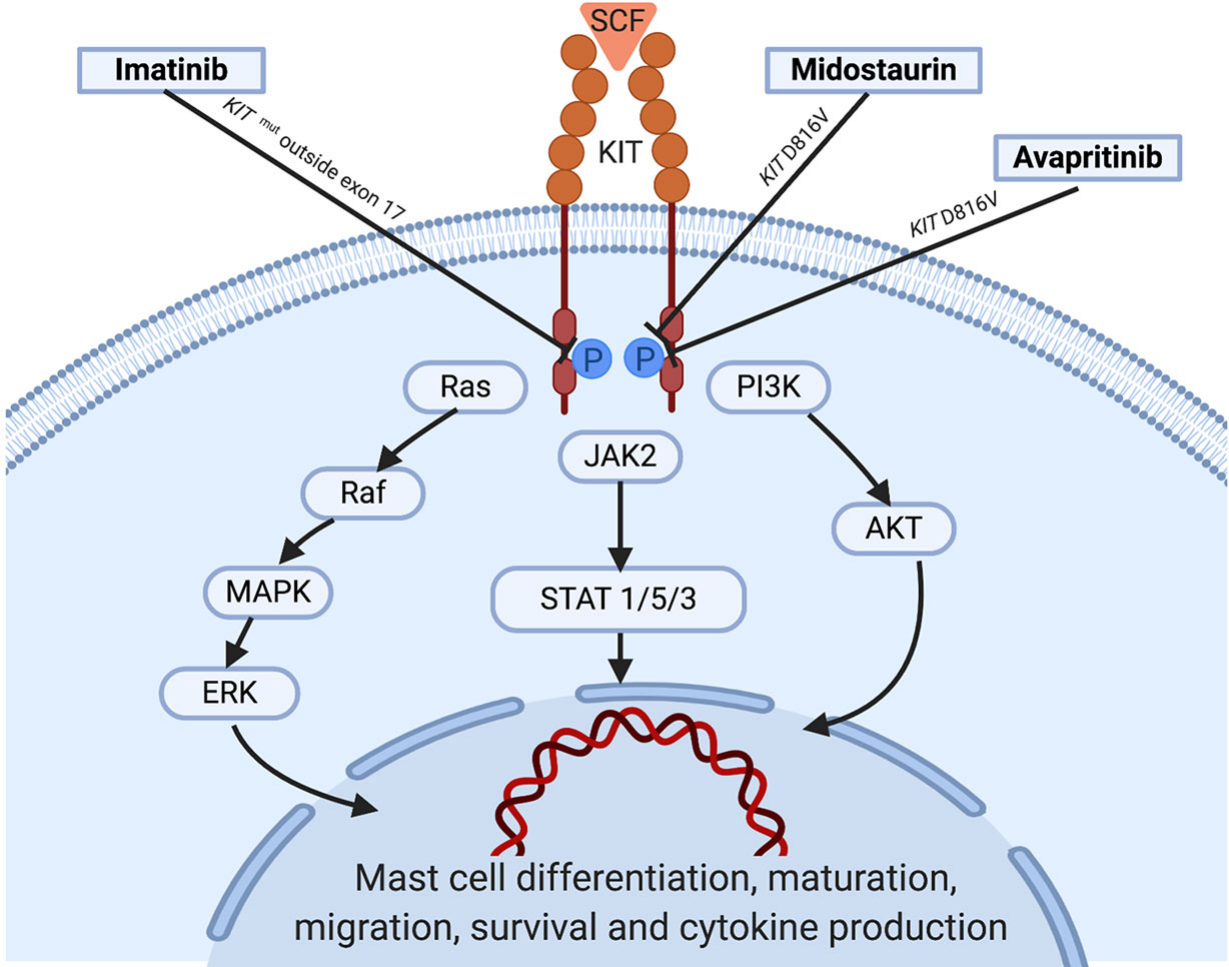
KIT activation in normal mast cells: Under normal conditions, soluble SCF binds to *KIT* leading to receptor dimerization and kinase domain activation, which induces the initiation of a cascade of multimolecular phosphorylation events involving a variety of intracellular signal transduction pathways such as the phosphatydylinositol triphosphate kinase (PI3K) pathway, the Janus kinase (JAK) / signal transducers and activators of transcription (STAT) pathway, and the rat sarcoma (Ras)/extracellular signal-regulated kinases (ERK) pathway (Orfao et al., 2007; Cruse et al., 2014; Grinfeld et al., 2018), among others. In parallel with the complex process underlying *KIT* activation, strict regulatory mechanisms including the monoubiquitination of *KIT* that occurs after *KIT*/SCF binding and the action of inhibitory molecules such as SHP-1, PKC, or SOCS-1 play an important role in hampering exaggerated and potentially harmful activation states of the receptor. Information about the targets of the tyrosine kinase inhibitors showing activity is illustrated. SCF, stem cell factor; WT, wild type.

MCs are key players in allergy and inflammatory response that derive from a hematopoietic progenitor cell arising in the bone marrow (Kirshenbaum et al., 1991). After early partial differentiation in the BM, MCs are released still as MC precursors into the bloodstream, from which they spread to peripheral organs and tissues, where they complete their maturation and differentiation *via* SCF-regulated *KIT* activation (Okayama and Kawakami, 2006). KIT is a member of the type III TK receptors which plays a central role in the control of differentiation, growth and survival of MCs (**Figure 1**). Structurally, KIT contains an extracellular domain with five immunoglobulin-like motifs that constitutes the SCF-binding site, a transmembrane domain, a juxtamembrane domain and two catalytic, functionally active kinase domains separated by a kinase insert (Cruse et al., 2014).

In mastocytosis, KIT is constitutively activated, which leads to persistent downstream activation signaling. The mechanism of constitutive activation of KIT is explained in >90% of patients with SM by the existence of somatic activating point mutations located at exon 17 of *KIT*, where resides the catalytic domain of the receptor (Kristensen et al., 2014; Jara-Acevedo et al., 2015). The most frequent *KIT* mutation found in SM is the D816V *KIT* mutation, which consists of the replacement of aspartic acid by valine in position 816 of the protein receptor (Nagata et al., 1995). Mutations other than the D816V *KIT* mutation have been rarely reported in SM, particularly in patients with a biological variant of the disease known as well-differentiated SM (WDSM) (Georgin-Lavialle et al., 2013; Arock et al., 2015; García-Montero et al., 2015).

### Classification and Prognostic Stratification of Mastocytosis

According to the World Health Organization (WHO), mastocytosis has been classically classified within the category of myeloproliferative neoplasms; however, in the 2016 update of the WHO classification of tumors of hematopoietic and lymphoid tissues, mastocytosis qualifies as a separate category within myeloid neoplasms (Arber et al., 2016). Despite this, diagnostic criteria for mastocytosis remain unchanged compared to previous versions of the WHO classification. Thus, diagnosis of cutaneous mastocytosis (CM) requires the presence of typical skin lesions together with the histological demonstration of abnormal MC infiltration of the dermis in the absence of criteria for SM. In turn, the diagnosis of SM is based on well-defined diagnostic criteria which include one major criterion and four minor criteria. The major criterion consists of multifocal dense infiltrates of ≥15 MCs in biopsy sections of BM and/or other extracutaneous organ(s) while the minor criteria include: 1) abnormal morphology of MCs from BM or other extracutaneous organ(s), 2) aberrant expression of CD25 (with or without CD2) in MCs from BM, blood or other extracutaneous organ(s), 3) activating point mutation at codon 816 of *KIT* in BM, blood or other extracutaneous organ(s), and 4) serum tryptase persistently >20 μg/L (Arber et al., 2016). The diagnosis of SM is established when the major criterion and at least one of the minor criteria, or when ≥3 minor criteria are fulfilled. Based on these criteria, together with the presence vs. absence of clinical and biological findings, associated haematological neoplasms, and the extension of BM MC involvement, SM can be subclassified into 5 variants: 1) indolent SM (ISM), 2) smouldering SM (SSM), 3) SM with an associated haematological neoplasm (SM-AHN), 4) aggressive SM (ASM), and 5) MC leukemia (MCL).

The prognostic impact of the WHO classification has been widely demonstrated in clinical studies showing that overall survival (OS) of patients with ASM, SM-AHN and MCL is significantly shortened; accordingly, these three variants of SM have been called “advanced SM” (AdvSM) (Lim et al., 2009). On the contrary, the life expectancy of patients with CM and ISM is similar to that of normal individuals. (Lim et al., 2009). In turn, SSM is currently considered as an intermediate-prognosis variant of SM, with better OS and progression-free survival than AdvSM but worse than ISM (Valent et al., 2002).

Despite its utility in the prognostic stratification of patients with SM, the current WHO classification of mastocytosis fails to identify a subgroup of patients with ISM who will eventually end up developing AdvSM. Previous studies have shown that the detection of the D816V *KIT* mutation not only in BM MCs but also in other myeloid or myeloid plus lymphoid BM cells and the demonstration of increased serum β_2_-microglobulin levels at diagnosis constitute the best combination of predictive factors for clinical progression in ISM (Escribano et al., 2009). By contrast, a few patients with AdvSM show a more favorable clinical behavior with prolonged survival rates. Recent investigations have suggested that the absence of mutations in genes other than *KIT* including *SRSF2*, *ASXL1*, *RUNX1*, and *EZH2* might be associated with better prognosis in terms of OS in patients with AdvSM (Jawhar et al., 2016; Muñoz-González et al., 2018).

In addition, the existence of atypical mutations in specific regions of *KIT* may translate into prognostic implications. Thus, in contrast to patients with the typical D816V *KIT* mutation who are intrinsically resistant to imatinib, this TKI is able to induce complete and maintained remissions in patients showing mutations outside the catalytic domain of *KIT*, mostly those involving exons 8–11 which are particularly frequent in WDSM (Valent et al., ; Vega-Ruiz et al., 2009; Mital et al., 2011; de Melo Campos et al., 2014; García-Montero et al., 2015; Alvarez-Twose et al., 2017; Broderick et al., 2019).

Altogether, these observations support the need for implementation of extended genetic analyses beyond the study of the D816V *KIT* mutation in the diagnostic work-up of mastocytosis which could help to select for those patients with AdvSM who will require more aggressive therapies, and to identify a subgroup of patients with ISM at risk of progression for whom a closer follow-up should be recommended. Furthermore, in the few patients with proven SM lacking the D816V *KIT* mutation, sequencing of the whole *KIT* gene becomes essential for a clear distinction between patients who are candidates for treatment with imatinib (e.g. patients with mutations in exons 8–11) and those who are imatinib-resistant (e.g. patients with other mutations in exon 17).

### Critical Factors and Limitations for the Selection of Cytoreductive Therapies in Mastocytosis

At present, mastocytosis is considered an incurable disease. Although most patients require anti-mediator therapies in order to prevent and/or minimize MC activation-related symptoms, only a minority of patients with SM are potential candidates for cytoreductive drugs. The most important factors to keep in mind when it comes to deciding on any of the currently available cytoreductive therapies for mastocytosis include the subtype of SM, the expected effect in terms of MC cytoreduction of the selected treatment and its potential toxicity. In general, cytoreductive approaches are usually restricted to patients diagnosed with AdvSM (i.e. ASM, SM-AHN or MCL); much more rarely, cytoreduction might be recommended for a small subset of patients presenting with highly symptomatic ISM refractory to conventional anti-mediator drugs. The limitations for deciding which therapeutic approach would be most beneficial over others in every specific case derive from the rarity of AdvSM, the scarcity of prospective clinical studies, the lack of randomized controlled trials and the absence of homogeneous and widely accepted response criteria before 2007.

### Tyrosine Kinase Inhibitor Therapy in Mastocytosis

Before the emergence of TKIs, treatments most commonly used in AdvSM included interferon-alpha (IFN-α), cladribine, hydroxyurea and, in selected cases, hematopoietic stem cell transplantation (HSCT); however, except for isolated cases, none of these approaches have shown to induce significant and maintained responses in terms of reduction of the neoplastic MC burden in AdvSM. Moreover, the available data on the efficacy of these treatments in patients with mastocytosis are mostly based on case reports, short patient series and retrospective studies (Lim et al., 2009; Barete et al., 2015). The discovery of the D816V *KIT* mutation as a pathogenic hallmark of SM in the last decade of XX century (Nagata et al., 1995), together with the development of the first drugs targeting TK-mediated signaling pathways a few years later have resulted into the beginning of a new era in the treatment of SM. An overview of the different TKIs investigated in SM is depicted in **Table 1**.

**Table 1:** Overview of main tyrosine-kinase inhibitor drugs investigated in patients with advanced systemic mastocytosis.

TKI	Number of AdvSM reported (ref)	Activity in SM	Activity in KIT D816V	Current status
**Imatinib**	32 (Lim et al., 2009; Vega-Ruiz et al., 2009; Alvarez-Twose et al., 2017)	High in sensitive mutations (KIT outside exon 17 or PDGFR)	-	FDA approved for Adult patients ASM without the D816V *KIT* or unknown KIT mutational status
**Nilotinib**	44 (Hochhaus et al., 2015)	Low	-/+	Inactive
**Dasatinib**	19 (Purtill et al., 2008; Verstovsek et al., 2008)	Low	-	Inactive
**Masitinib**	–	Modest in ISM with related handicap	-	Under investigation in phase 3 trials in severe ISM and SSM with related handicap
**Midostaurin**	142 (Gotlib et al., 2016; DeAngelo et al., 2018)	High (60-69% ORR)	++	Approved by the FDA and the EMA for AdvSM
**Avapritinib**	24 (Deininger et al., 2018)	High (83% ORR)	+++	Under investigation in phase 2 trials in AdvSM and in ISM and SSM with bad symptom control

ASM, aggressive systemic mastocytosis; ISM, indolent systemic mastocytosis; SSM, smouldering systemic mastocytosis; ORR, overall rate response.

#### Imatinib

The first TKI developed for clinical use in humans was imatinib mesylate, which was initially conceived as a specific *ABL-BCR* fusion protein inhibitor for patients with CML. Early *in vitro* and *in vivo* studies showed a marked ability of imatinib to kill CML cells by competitively binding to the ATP binding site of the *ABL* kinase domain, which paved the way for a dramatic change in the management and prognosis of CML (Druker et al., 2001). Beyond the outstanding results obtained in CML, imatinib also showed activity against other TKs such as *PDGFR* and *KIT*, which led to explore its efficacy in diseases driven by genetic alterations in these receptors including mastocytosis (Akin et al., 2003). However, patients with SM carrying the typical D816V *KIT* mutation show an intrinsic resistance to imatinib therapy due to a conformational change in the enzymatic pocket that blocks the binding of the drug to the receptor (Laine et al., 2011); in turn, imatinib have shown to inhibit the growth of MCs with wild-type *KIT* or with mutations outside the activation loop of *KIT* such as V560G, F522C, K509I, or p.419del *KIT* mutations (Frost et al., 2002; de Melo Campos et al., 2014; Broderick et al., 2019). Clinical data from single case reports and small series of patients with mastocytosis treated with imatinib led the U.S. Food and Drug Administration (FDA), but not the European Medicines Agency (EMA), to approve the drug in 2006 for adult patients with ASM without the D816V *KIT* mutation or with unknown or unavailable *KIT* mutational status. More recently, a clinical trial carried out by the Spanish Network on Mastocytosis (REMA) showed response to imatinib in 5/10 patients with SM lacking exon 17 *KIT* mutations, which included three WDSM patients with the K509I *KIT* mutation, one patient with wild-type *KIT* SM-chronic eosinophilic leukemia who had no *PDGFR* rearrangements and 1 patient with wild-type *KIT* WDSM (Alvarez-Twose et al., 2017). These observations together with data from a critical systematic review of all cases of mastocytosis treated with imatinib published in the literature by that time (n=121) support that response to imatinib in SM patients heavily relies on the presence of imatinib-sensitive mutations either involving *KIT* (e.g. juxtamembrane or transmembrane *KIT* mutations) or *PDGFR* (e.g. *FIP1L1*/*PDGFRα* rearrangement) rather than on the absence of the D816V *KIT* mutation (Alvarez-Twose et al., 2017). On the other hand, response to imatinib in terms of significant MC cytoreduction (i.e. ≥50%) in those patients who are not screened for the *KIT* mutation in the absence of imatinib-sensitive mutations involving other genes (e.g. *PDGFR*) is anecdotal (i.e. 3%) (Alvarez-Twose et al., 2017), which highlights the relevance of the study of the *KIT* mutational status before selecting potential candidates to imatinib therapy among patients with mastocytosis.

#### Nilotinib

Nilotinib is a second generation TKI which was rationally designed to overcome resistance to imatinib in CML. Besides inhibition of *BCR-ABL*, nilotinib has also shown *in vitro* activity against other kinases, particularly *PDGFRα* and *KIT*, which has led to the investigation of its potential clinical utility in diseases driven by these kinases such as gastrointestinal stromal tumors (GISTs) and mastocytosis. In a phase 2 open-label clinical trial including 37 patients with ASM who received nilotinib, the overall response rate observed in this group of patients was 21.6%, which mostly consisted of modest responses in terms of BM MC cytoreduction; nevertheless, all these partial responses were seen in patients carrying the typically imatinib-resistant D816V *KIT* mutation (Hochhaus et al., 2015).

#### Dasatinib

Dasatinib is another second generation *BCR-ABL* inhibitor that has also been proven to display an inhibitory effect *in vitro* against other TKs including *KIT* (Tokarski et al., 2006). Similarly to imatinib and nilotinib, dasatinib has shown to be effective in patients with CML, but its activity in patients with SM seems to be limited. A phase II clinical study of dasatinib in a group of 33 patients with SM including nine ASM, 18 ISM, and six SM-AHN showed an overall response rate of 33% (Verstovsek et al., 2008). There were two patients with D816V-negative SM-AHN who achieved complete response after dasatinib therapy, whereas the remaining nine responding patients showed only symptomatic improvement.

#### Masitinib

Masitinib is a multi-targeted protein kinase inhibitor with activity *in vitro* against *PDGFR*, *Lyn*, *Fyn*, and wild-type *KIT* but not against D816V-mutated *KIT* (Dubreuil et al., 2009). Clinical studies of masitinib in mastocytosis patients are mainly focused on exploring its potential utility for MC-mediator associated symptoms. Thus, a phase 2 trial in 25 patients with CM and SM with related handicap (i.e. disabilities associated with flushes, pruritus, depression and quality of life) showed an overall symptomatic response in 56% of patients (Paul et al., 2010). However, a phase 3 randomized, placebo-controlled study in 135 patients with severely symptomatic ISM revealed a modest efficacy of masitinib for the control of pruritus, flushing, depression or fatigue, with an overall cumulative response rate of 18.7% vs. 7.4% in the placebo arm (Lortholary et al., 2017).

#### Midostaurin

Midostaurin is a staurosporine-derived multikinase inhibitor that targets mutant forms of Fms-like TK3 (*FLT3*), both wild-type and D816V-mutated *KIT*, as well as additional protein kinases such as kinase insert domain-containing receptor (*KDR*), fibroblast growth factor receptor (*FGFR*), vascular endothelial growth factor receptor 2 (*VEGFR2*), *FIP1L1/PDGFRα* fusion protein, and members of the serine/threonine protein kinase C (*PKC*) family (Fabrro et al., 2000). Like imatinib, midostaurin competitively binds to the ATP binding site in the catalytic domain of TKs, which results in their inhibition. Although early preclinical studies suggested that midostaurin might be useful in a wide variety of TK-driven malignant diseases including solid tumors and haematological neoplasms (Monnerat, 2004; Millward et al., 2006), a relevant clinical efficacy has only been demonstrated in *FLT3*-positive acute myeloid leukemia (AML) and AdvSM, which actually constitute the only two indications of midostaurin approved by the U.S. FDA and the EMA. Regarding *KIT* inhibition, midostaurin has been found to display synergistic growth-inhibitory effects on neoplastic D816V-positive MCs in combination with other TKIs such as ponatinib and dasatinib (Gleixner et al., 2007; Gleixner et al., 2013).

The pivotal clinical study in mastocytosis that led to drug approval for this indication was a single-arm, phase 2 trial in 89 patients with ASM (n=16), SM-AHN (n=57) or MCL (n=16), mostly carrying the D816V *KIT* mutation (Gotlib et al., 2016). The overall response rate in terms of reduction in MC burden was of 60%, with a median OS of 28.7 months, which was significantly higher in responders vs. non-responders (44 vs. 15 months, respectively). Notably, when response rate is analyzed depending on the specific subtype of SM, patients with ASM appear to show higher response rate and longer OS vs. MCL and SM-AHN patients. In contrast to clinical data in mastocytosis patients treated with other TKIs such as imatinib, the response rate to midostaurin in D816V-positive AdvSM was even higher than that found in patients with wild-type *KIT* or unknown *KIT* mutational status (63% vs. 44%), which is consistent with the results of prior *in vitro* investigations showing that midostaurin potently inhibits the imatinib-resistant D816V- and D816Y-mutated *KIT* forms (Gleixner, 2006). More recently published long-term results from a phase 2, investigator-initiated trial in 26 patients with SM (three ASM, 17 SM-AHN, and six MCL) receiving midostaurin for a median of 10 years have shown an overall response rate during the first year of treatment of 69%, with a median OS of 40 months (DeAngelo et al., 2018). Interestingly, two patients fulfilled criteria for complete remission when they were evaluated for response beyond 12 months of therapy (at time check-points of 24 and 30 months of follow up, respectively). Another recent study in 38 patients with AdvSM who received treatment with midostaurin has shown that a decrease ≤ 25% of the D816V *KIT* allele burden and the presence of additional mutations in genes other than *KIT* (e.g. *SRSF2*, *ASXL1*, and *RUNX1*) are indicators of a poorer outcome (Jawhar et al., 2017).

Despite the lack of randomized studies of midostaurin in AdvSM, a recent study comparing the outcome of patients included in the pivotal trial of midostaurin detailed above with data obtained from the German registry of AdvSM including a historical cohort of 46 patients treated with therapies other than midostaurin, revealed two-fold increase in OS in the group of patients treated with midostaurin (41.4 vs. 19.5 months) (Reiter et al., 2017).

From a purely clinical point of view, midostaurin was also able to improve virtually all mastocytosis-related symptoms in patients with AdvSM included in the aforementioned trials, which has led to explore its potential utility also in severely symptomatic non-advanced SM refractory to conventional antimediator therapies. In a phase 2 study in 20 ISM patients, midostaurin showed improvement of symptoms in 75% of cases and a median 29% improvement of quality of life with favorable tolerability profile (van Anrooij et al., 2018).

#### Novel TKIs

In recent years, novel TKIs with the ability of binding KIT more specifically than their predecessors have been developed. A phase 1 trial of avapritinib in patients with AdvSM has already shown promising results including an overall response rate of 72% after 9 months of therapy and the demonstration of dramatic and durable reductions in both MC burden and D816V *KIT* allele fraction (Deininger et al., 2018); moreover, avapritinib has proven to show activity *in vitro* in midostaurin-resistant MCs (Lübke et al., 2019). At present, a multicenter phase 2 single-arm study to evaluate the efficacy and safety of avapritinib in AdvSM patients is ongoing. DCC-2618 is also a new potent multi-targeted TKI which has shown a potent inhibitory effect on the growth and survival of neoplastic MCs *in vitro* for which clinical studies are currently being conducted (Schneeweiss et al., 2018).

### Personal Approach

**Figure 2:**
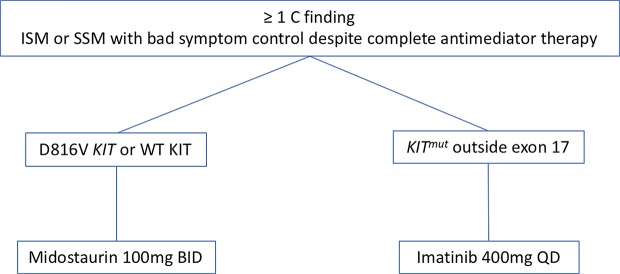
Therapeutic algorithm with TKIs. Abbreviations: TKIs, TK inhibitors; ISM, indolent systemic mastocytosis; SSM, smouldering systemic mastocytosis.

Based on the aforementioned data we focus on the *KIT* mutational status when we face a patient with AdvSM (**Figure 2**). Thus, once we consider cytoreductive therapy is needed (see above) and taking into consideration that the *KIT* mutational status must be performed with highly-sensible techniques in order to reliably consider a patient as *KIT* WT, we prefer to initiate TKIs as first line therapy in most cases. In cases with KIT mutation outside exon 17, we start treatment with Imatinib 400 mg QD. In cases with *KIT* mutations in exon 17 or *KIT* WT, we choose midostaurin 100 mg BID as first line therapy. In relapsed/refractory patients, new TKIs such as avapritinib have shown promising activity with an acceptable safety profile, seeming a good approach. However, more data from the phase 2 trails are still needed in order to make a formal recommendation. In this relapsed/refractory setting, we also consider therapy with cladribine or with pegylated-interferon.

## Conclusion

SM is a rare, heterogeneous disease whose pathogenesis is driven by *KIT* mutations. The intrinsic resistance to the first generation TKI imatinib conferred by the typical D816V *KIT* mutation, has led to explore the potential utility of novel, highly selective TKIs such as midostaurin and avapritinib, which have demonstrated activity against *KIT* D816V forms of SM. The emergence of these improved targeted therapies has paved the way for a new era in the management of patients with advanced SM.

## Author Contributions

MP-V made the design and the draft. IA-T made the revision.

## Funding

This work was supported by grants from the Asociación Española de Mastocitosis y enfermedades relacionadas (AEDM 2017, Madrid, Spain) and from Hospital Virgen de la Salud Biobank (BioB-HVS), supported by grant PT13/0010/0007 from the Instituto de Salud Carlos III, Madrid, Spain.

## Conflict of Interest

MP-V and IA-T have a consultancy agreement with Novartis.

## References

[B1] Akin C. Brockow K. D’Ambrosio C. Kirshenbaum A. S. Ma Y. Longley B. J. (2003). Effects of tyrosine kinase inhibitor STI571 on human mast cells bearing wild-type or mutated c-kit. Exp. Hematol. 31 (8), 686–692. 10.1016/S0301-472X(03)00112-7 12901973

[B2] Alvarez-Twose I. Matito A. Morgado J. M. Muñoz L.--. Jara-Acevedo M. García-Montero A. (2017). Imatinib in systemic mastocytosis: a phase IV clinical trial in patients lacking exon 17 *KIT* mutations and review of the literature. Oncotarget. Sep 15 [cited 2019 Apr 14]; 8 (40), 68950–68963. Available from: http://www.oncotarget.com/fulltext/10711. 28978170 10.18632/oncotarget.10711 PMC5620310

[B3] Arber D. A. Orazi A. Hasserjian R. Thiele J. Borowitz M. J. Le Beau M. M. (2016). The 2016 revision to the World Health Organization classification of myeloid neoplasms and acute leukemia. Blood. 127 (20), 2391–2405. 10.1182/blood-2016-03-643544 27069254

[B4] Arock M. Sotlar K. Akin C. Broesby-Olsen S. Hoermann G. Escribano L. (2015). KIT mutation analysis in mast cell neoplasms: Recommendations of the European Competence Network on Mastocytosis. Leukemia 29 (6), 1223–1232. 10.1038/leu.2015.24 25650093 PMC4522520

[B5] Barete S. Lortholary O. Damaj G. Hirsch I. Chandesris M. O. Elie C. (2015). Long-term efficacy and safety of cladribine (2-CdA) in adult patients with mastocytosis. Blood. 126 (8), 1009–1016. 10.1182/blood-2014-12-614743 26002962

[B6] Broderick V. Waghorn K. Langabeer S. E. Jeffers M. Cross N. C. P. Hayden P. J. (2019). Molecular response to imatinib in KIT F522C-mutated systemic mastocytosis. Leuk Res. 77, 28–29. 10.1016/j.leukres.2018.12.010 30612056

[B7] Cruse G. Metcalfe D. D. Olivera A. (2014). Functional deregulation of KIT: Link to mast cell proliferative diseases and other neoplasms. Immunol. Allergy Clin. North Am. 34 (2), 219–237. 10.1016/j.iac.2014.01.002 24745671 PMC3994404

[B8] de Melo Campos P. Machado-Neto J. A. Scopim-Ribeiro R. Visconte V. Tabarroki A. Duarte A. S. S. (2014). Familial systemic mastocytosis with germline KIT K509I mutation is sensitive to treatment with imatinib, dasatinib and PKC412. Leuk Res. 38 (10), 1245–1251. 10.1016/j.leukres.2014.07.010 25139846

[B9] DeAngelo D. J. George T. I. Linder A. Langford C. Perkins C. Ma J. (2018). Efficacy and safety of midostaurin in patients with advanced systemic mastocytosis: 10-year median follow-up of a phase II trial. Leukemia. 32 (2), 470–478. 10.1038/leu.2017.234 28744009

[B10] Deininger M. Gotlib J. Robinson W. A. Radia D. H. Drummond M. W. Quiery A. T. (2018). Avapritinib (blu285), a selective kit inhibitor, is associated with high response rate and tolerable safety profile in advanced systemic mastocytosis (advsm): results of a phase 1 study 3 EHA Annual Meeting, 2018. Abstract PF612

[B11] Druker B. J. Talpaz M. Resta D. J. Peng B. Buchdunger E. Ford J. M. (2001). Efficacy and Safety of a Specific Inhibitor of the BCR-ABL Tyrosine Kinase in Chronic Myeloid Leukemia. N Engl. J. Med. 344 (14), 1031–1037. 10.1056/NEJM200104053441401 11287972

[B12] Dubreuil P. Letard S. Ciufolini M. Gros L. Humbert M. Castéran N. (2009). Masitinib (AB1010), a Potent and Selective Tyrosine Kinase Inhibitor Targeting KIT. PLoS ONE 4 (9): e7258. 10.1371/journal.pone.0007258 19789626 PMC2746281

[B13] Escribano L. Álvarez-Twose I. Sánchez-Muñoz L. Garcia-Montero A. Núñez R. Almeida J. (2009). Prognosis in adult indolent systemic mastocytosis: A long-term study of the Spanish Network on Mastocytosis in a series of 145 patients. J. Allergy Clin. Immunol. 124 (3), 514–521. 10.1016/j.jaci.2009.05.003 19541349

[B14] Fabrro D. Ruetz S. Bodis S. Pruschy M. Csermak K. Man A. (2000). PKC412–a protein kinase inhibitor with a broad therapeutic potential. Anticancer Drug Des. 15, 17–28. 10888033

[B15] Frost M. J. Ferrao P. T. Hughes T. P. Ashman L. K. (2002). Juxtamembrane Mutant V560GKit Is More Sensitive to Imatinib (STI571) Compared with Wild-Type c-Kit Whereas the Kinase Domain Mutant D816VKit Is Resistant 11. Mol. Cancer Ther. 1, 1115–1124. 12481435

[B16] García-Montero A. Álvarez-Twose I. Mayado A. Mollejo M. Sánchez-Muñoz L. Jara-Acevedo M. (2015). Clinical, immunophenotypic, and molecular characteristics of well-differentiated systemic mastocytosis. J. Allergy Clin. Immunol. 137 (1), 168–178.e1. 10.1016/j.jaci.2015.05.008 26100086

[B17] Garcia-Montero A. C. Jara-Acevedo M. Teodosio C. Sanchez M. L. Nunez R. Prados A. (2006). KIT mutation in mast cells and other bone marrow hematopoietic cell lineages in systemic mast cell disorders: A prospective study of the Spanish Network on Mastocytosis (REMA) in a series of 113 patients. Blood 108 (7), 2366–2372. 10.1182/blood-2006-04-015545 16741248

[B18] Georgin-Lavialle S. Lhermitte L. Dubreuil P. Chandesris M. O. Hermine O. Damaj G. (2013). Mast cell leukemia. Blood. 121 (8), 1285–1295. 10.1182/blood-2012-07-442400 23243287

[B19] Gleixner K. V. Mayerhofer M. Sonneck K. Gruze A. Samorapoompichit P. Baumgartner C. (2007). Synergistic growth-inhibitory effects of two tyrosine kinase inhibitors, dasatinib and PKC412, on neoplastic mast cells expressing the D816V-mutated oncogenic variant of KIT. Haematologica. 92 (11), 1451–1459. 10.3324/haematol.11339 18024392

[B20] Gleixner K. V. Peter B. Blatt K. Suppan V. Reiter A. Radia D. (2013). Synergistic growth-inhibitory effects of ponatinib and midostaurin (PKC412) on neoplastic mast cells carrying KIT D816V. Haematologica. 98 (9), 1450–1457. 10.3324/haematol.2012.079202 23539538 PMC3762103

[B21] Gleixner K. V. (2006). PKC412 inhibits in vitro growth of neoplastic human mast cells expressing the D816V-mutated variant of KIT: comparison with AMN107, imatinib, and cladribine (2CdA) and evaluation of cooperative drug effects. Blood. 107 (2), 752–759. 10.1182/blood-2005-07-3022 16189265

[B22] Gotlib J. Kluin-Nelemans H. C. George T. I. Akin C. Sotlar K. Hermine O. (2016). Efficacy and Safety of Midostaurin in Advanced Systemic Mastocytosis. N Engl. J. Med. 374 (26), 2530–2541. 10.1056/NEJMoa1513098 27355533

[B23] Grinfeld J. Nangalia J. Baxter E. J. Wedge D. C. Angelopoulos N. Cantrill R. (2018). Classification and Personalized Prognosis in Myeloproliferative Neoplasms. N Engl. J. Med. 379 (15), 1416–1430. 10.1056/NEJMoa1716614 30304655 PMC7030948

[B24] Hochhaus A. Baccarani M. Giles F. J. le Coutre P. D. Müller M. C. Reiter A. (2015). Nilotinib in patients with systemic mastocytosis: analysis of the phase 2, open-label, single-arm nilotinib registration study. J. Cancer Res. Clin. Oncol. 141, 2047–2060. 10.1007/s00432-015-1988-0 26002753 PMC4768228

[B25] Jara-Acevedo M. Teodosio C. Sanchez-Muñoz L. Álvarez-Twose I. Mayado A. Caldas C. (2015). Detection of the KIT D816V mutation in peripheral blood of systemic mastocytosis: diagnostic implications. Mod Pathol. 28 (8), 1138–1149. 10.1038/modpathol.2015.72 26067933

[B26] Jawhar M. Schwaab J. Schnittger S. Meggendorfer M. Pfirrmann M. Sotlar K. (2016). Additional mutations in SRSF2, ASXL1 and/or RUNX1 identify a high-risk group of patients with KIT D816V+ advanced systemic mastocytosis. Leukemia. 30 (1), 136–143. 10.1038/leu.2015.284 26464169

[B27] Jawhar M. Schwaab J. Naumann N. Horny H.-P. Sotlar K. Haferlach T. (2017). Response and progression on midostaurin in advanced systemic mastocytosis: *KIT* D816V and other molecular markers. Blood 130 (2), 137–145. 10.1182/blood-2017-01-764423 28424161

[B28] Kirshenbaum A. S. Kessler S. W. Goff J. P. Metcalfe D. D. (1991). Demonstration of the origin of human mast cells from CD34+ bone marrow progenitor cells. J. Immunol. Baltim Md 1950 146 (5), 1410–1415. 1704394

[B29] Kristensen T. Vestergaard H. Bindslev-Jensen C. Møller M. B. Broesby-Olsen S. (2014). Sensitive KIT D816V mutation analysis of blood as a diagnostic test in mastocytosis. Am. J. Hematol. 89 (5), 493–498. 10.1002/ajh.23672 24443360

[B30] Laine E. Chauvot de Beauchêne I. Perahia D. Auclair C. Tchertanov L. (2011). Mutation D816V Alters the Internal Structure and Dynamics of c-KIT Receptor Cytoplasmic Region: Implications for Dimerization and Activation Mechanisms. Verkhivker GM editor. PloS Comput. Biol. 7 (6), e1002068. 10.1371/journal.pcbi.1002068 PMC3116893 21698178

[B31] Lim K. H. Tefferi A. Lasho T. L. Finke C. Patnaik M. Butterfield J. H. (2009). Systemic mastocytosis in 342 consecutive adults: Survival studies and prognostic factors. Blood 113 (23), 5727–5736. 10.1182/blood-2009-02-205237 19363219

[B32] Lim K. H. Pardanani A. Butterfield J. H. Li C.-Y. Tefferi A. (2009). Cytoreductive therapy in 108 adults with systemic mastocytosis: Outcome analysis and response prediction during treatment with interferon-alpha, hydroxyurea, imatinib mesylate or 2-chlorodeoxyadenosine. Am. J. Hematol. 84 (12), 790–794. 10.1002/ajh.21561 19890907

[B33] Lortholary O. Chandesris M. O. Livideanu C. B. Paul C. Guillet G. Jassem E. (2017). Masitinib for treatment of severely symptomatic indolent systemic mastocytosis: a randomised, placebo-controlled, phase 3 study. Lancet 389 (10069), 612–620. 10.1016/S0140-6736(16)31403-9 28069279 PMC5985971

[B34] Lübke J. Naumann N. Kluger S. Schwaab J. Metzgeroth G. Evans E. (2019). Inhibitory effects of midostaurin and avapritinib on myeloid progenitors derived from patients with KIT D816V positive advanced systemic mastocytosis. Leukemia. Mar 25 [cited 2019 Apr 17]; 33, 1195–1205. Available from: http://www.nature.com/articles/s41375-019-0450-8. 10.1038/s41375-019-0450-8 PMC6756065 30911112

[B35] Millward M. J. House C. Bowtell D. Webster L. Olver I. N. Gore M. (2006). The multikinase inhibitor midostaurin (PKC412A) lacks activity in metastatic melanoma: a phase IIA clinical and biologic study. Br. J. Cancer. 95 (7), 829–834. 10.1038/sj.bjc.6603331 16969355 PMC2360547

[B36] Mital A. Piskorz A. Lewandowski K. Wasąg B. Limon J. Hellmann A. (2011). A case of mast cell leukaemia with exon 9 KIT mutation and good response to imatinib: Mast cell leukemia with KIT mutation. Eur. J. Haematol. 86 (6), 531–535. 10.1111/j.1600-0609.2011.01598.x 21362052

[B37] Monnerat C. (2004). Phase I study of PKC412 (N-benzoyl-staurosporine), a novel oral protein kinase C inhibitor, combined with gemcitabine and cisplatin in patients with non-small-cell lung cancer. Ann. Oncol. 15 (2), 316–323. 10.1093/annonc/mdh052 14760128

[B38] Muñoz-González J. I. Jara-Acevedo M. Alvarez-Twose I. Merker J. D. Teodosio C. Hou Y. (2018). Impact of somatic and germline mutations on the outcome of systemic mastocytosis. Blood Adv. 2 (21), 2814–2828. 10.1182/bloodadvances.2018020628 30373888 PMC6234367

[B39] Nagata H. Worobec A. S. Oh C. K. Chowdhury B. A. Tannenbaum S. Suzuki Y. (1995). Identification of a point mutation in the catalytic domain of the protooncogene c-kit in peripheral blood mononuclear cells of patients who have mastocytosis with an associated hematologic disorder. Proc. Natl. Acad. Sci. 92 (23), 10560–10564. 10.1073/pnas.92.23.10560 7479840 PMC40651

[B40] Okayama Y. Kawakami T. (2006). Development, Migration, and Survival of Mast Cells. Immunol. Res. 34 (2), 97–115. 10.1385/IR:34:2:97 16760571 PMC1490026

[B41] Orfao A. Garcia-Montero A. C. Sanchez L. Escribano L. (2007). Recent advances in the understanding of mastocytosis: The role of KIT mutations. Br. J. Haematol. 138 (1), 12–30. 10.1111/j.1365-2141.2007.06619.x 17555444

[B42] Paul C. Sans B. Suarez F. Casassus P. Barete S. Lanternier F. (2010). Masitinib for the treatment of systemic and cutaneous mastocytosis with handicap: A phase 2a study. Am. J. Hematol. 85 (12), 921–925. 10.1002/ajh.21894 21108325

[B43] Purtill D. Cooney J. Sinniah R. Carnley B. Cull G. Augustson B. (2008). Dasatinib therapy for systemic mastocytosis: four cases. Eur. J. Haematol. 80 (5), 456–458. 10.1111/j.1600-0609.2008.01048.x 18284618

[B44] Reiter A Kluin-Nelemans H. C. George T. Akin C. DeAngelo D. J. Hermine O. (2017). Pooled Survival Analysis Of Midostaurin Clinical Study Data (D2201 + A2213) In Patients With Advanced Systemic Mastocytosis (Advsm) Compared With Historical Controls. EHA annual meeting 2017, Abstract S788.. Available from: https://learningcenter.ehaweb.org/eha/2017/22nd/182075/andreas.reiter.pooled.survival.analysis.of.midostaurin.clinical.study.data.html.

[B45] Schneeweiss M. Peter B. Bibi S. Eisenwort G. Smiljkovic D. Blatt K. (2018). The KIT and PDGFRA switch-control inhibitor DCC-2618 blocks growth and survival of multiple neoplastic cell types in advanced mastocytosis. Haematologica. 103 (5), 799–809. 10.3324/haematol.2017.179895 29439183 PMC5927976

[B46] Tokarski J. S. Newitt J. A. Chang C. Y. J. Cheng J. D. Wittekind M. Kiefer S. E. (2006). The Structure of Dasatinib (BMS-354825) Bound to Activated ABL Kinase Domain Elucidates Its Inhibitory Activity against Imatinib-Resistant ABL Mutants. Cancer Res. 66 (11), 5790–5797. 10.1158/0008-5472.CAN-05-4187 16740718

[B47] Valent P. Cerny-Reiterer S. Hoermann G. Sperr W. R. Müllauer L. Mannhalter C. Long-lasting complete response to imatinib in a patient with systemic mastocytosis exhibiting wild type KIT 8. PMC4351647 25755909

[B48] Valent P. Akin C. Sperr W. R. Horny H.-P. Metcalfe D. D. (2002). Smouldering Mastocytosis: A Novel Subtype of Systemic Mastocytosis with Slow Progression. Int. Arch. Allergy Immunol. 127 (2), 137–139. 10.1159/000048185 11919424

[B49] van Anrooij B. Oude Elberink J. N. G. Span L. F. R. de Monchy J. G. R. Rosati S. Mulder A. B. (2018). Midostaurin in patients with indolent systemic mastocytosis: An open-label phase 2 trial. J. Allergy Clin. Immunol. 142 (3), 1006–1008.e7. 10.1016/j.jaci.2018.06.003 29890238

[B50] Vega-Ruiz A. Cortes J. E. Sever M. Manshouri T. Quintás-Cardama A. Luthra R. (2009). Phase II study of imatinib mesylate as therapy for patients with systemic mastocytosis. Leuk Res. 33 (11), 1481–1484. 10.1016/j.leukres.2008.12.020 19193436 PMC4184059

[B51] Verstovsek S. Tefferi A. Cortes J. O’Brien S. Garcia-Manero G. Pardanani A. (2008). Phase II Study of Dasatinib in Philadelphia Chromosome-Negative Acute and Chronic Myeloid Diseases, Including Systemic Mastocytosis. Clin. Cancer Res. 14 (12), 3906–3915. 10.1158/1078-0432.CCR-08-0366 18559612 PMC5018899

